# The impact of Yiwei decoction on the LncRNA and CircRNA regulatory networks in premature ovarian insufficiency

**DOI:** 10.1016/j.heliyon.2023.e20022

**Published:** 2023-09-09

**Authors:** Weisen Fan, Yingjie Zhang, Dandan Wang, Chen Wang, Jie Yang

**Affiliations:** a The First Clinical Medical College of Shandong University of Traditional Chinese Medicine, Jinan, 250013, China; b School of Health, Shandong University of Traditional Chinese Medicine, Jinan, 250013, China; c School of Traditional Chinese Medicine, Shandong University of Chinese Medicine, Jinan, 250013, China; d School of Physical Education and Health, Shandong Sport University, Jinan, 250013, China

**Keywords:** Transcriptomics, Traditional Chinese medicine, Premature ovarian insufficiency, Non-coding RNA, CeRNA

## Abstract

Premature ovarian insufficiency（POI）is a female reproductive aging illness. Yiwei decoction（YWD） is a traditional treatment for Yangming nourishment. YWD can treat premature ovarian insufficiency, but the exact molecular mechanism is unknown. As a result, the differential expression of Long noncoding RNAs (LncRNAs) and Circular RNAs(CircRNAs) in the ovary of POI rats after YWD treatment was investigated in this paper, and the CeRNA regulatory network was built.

The model was created using cyclophosphamide. The model group + YWD was in Group A, the model control group was in Group B, and the regular control group was in Group C. In this study, 177 differential expression Long noncoding RNAs(DELncRNAs) and 190 differential expression Circular RNAs (DECircRNAs) were discovered between A and B (P＜0.05,|LogFC|＞1). Following the analysis, 27 DELncRNAs and 96 DECircRNAs (P-adjusted＜0.05,|LogFC|＞1) were discovered. At the same time, we built the CeRNA network using differentially expressed mRNAs and microRNAs (miRNAs) expression between groups A and B. The DELncRNAs were used to construct a lncRNA-miRNA-mRNA ceRNA network with 27 LncRNAs, 4 miRNAs, and 19 mRNAs. The DECircRNAs were utilized to establish a CircRNA-miRNA-mRNA ceRNA network that was made up of 15 CircRNAs, 4 miRNAs, and 20 mRNA. The highly correlated regulatory networks were the LncMSTRG.22691.3/miR-3102/ANGPT4 and Circ10_34698898_34699378/miR-33-5p/TTC22. Circ20_12035276_12036793、Circ20_30693935_30696337、Circ4_157723097_157723378 and Circ4_157923266_157923904 occurred concurrently in AvsB, BvsC, and AvsC. MiRDB predicted eight target miRNAs for these CircRNAs. The miRanda(score = 140，energy = −1) binding energy calculation revealed that seven miRNAs were well combined with three CircRNA base complementary pairs. This implies that 3 DECircRNAs could serve as spongy bodies for these miRNAs. Network pharmacological analysis showed that ten active components in YWD may regulate the expression of LncRNAs and CircRNAs, such as Stigmasterol, Uridine, Ophiopogonanone A, Gamma-Aminobutyric Acid, and others.

In conclusion, this study combined transcriptomics and network pharmacological analysis to identify differentially expressed lncRNAs as well as CircRNAs in ovaries of YWD-treated POI rats, thereby constructing ceRNA networks implicated in POI. This would contribute to clarifying the pathways by which Chinese herbal compounds regulate gene expression in POI.

## 1 Introduction

Premature ovarian insufficiency(POI) is a problematic issue that inhibits female fertility. It is clinically manifested as decreased menstrual volume, amenorrhea, and infertility, and in severe cases, can progress to premature ovarian failure [1]. The treatment of this disease must be classified based on whether or not the patient requires fertility assistance. Hormone supplementation may be used for patients who do not need fertility treatment. The main goal of treating this disease for patients who require fertility treatment is to increase the dominant ovarian follicles [2,3]. Since ancient times, traditional Chinese medicine（TCM） has been praised for promoting women's reproductive health [4]. TCM effectively treats early-onset ovarian insufficiency, improves patients' ovarian reserve and lowers serum follicle-stimulating hormone, and increases estrogen levels [5,6]. There were records about "premature menstruation amenorrhea” in the Qing Dynasty, and most doctors began with the Angle of tonifying the kidney. However, according to Huangdi Neijing, female aging starts with lossing the Yangming pulse. However, according to Yellow Emperor's Internal Classic, female aging begins with the failure of the Yangming pulse. As a result, we chose the traditional prescription YWD to nourish Yang Ming.

Long noncoding RNA (LncRNA) and circular RNA(CircRNA) have recently become the focus of research due to their irreplaceable role in both the physiological and pathological conditions of cells [7]. LncRNAs are a type of RNA that has a length of more than 200 nucleotides and can regulate gene expression at multiple levels [8]. LncRNA action mechanisms can be divided into three categories [[9], [10], [11]]: a It has the potential to interfere with DNA expression directly. This effect is achieved by inhibiting RNA polymerase II and mediating chromatin remodeling and histone modification. b Binds to gene transcripts to form complementary double strands that disrupt mRNA splicing or generate endogenous siRNA. c It can bind to proteins to regulate protein activity or change cell localization. CircRNA is a non-coding RNA(ncRNA) molecule that exists in living organisms and has a ring structure with covalent bonds but no 5′cap and 3′poly(A) tail. Because of its closed ring structure, which is not easily degraded by exonuclease, CircRNA is more stable than linear RNA [12]. CircRNA can act as a sponge for proteins and miRNAs, binding to them and undoing both [13]. As a result, LncRNAs and CircRNAs can both regulate gene expression and play a role. Because YWD has a good effect in the treatment of POI, this paper investigated the expression of relevant lncRNAs and CircRNAs in the intervention of YWD in POI rats and analyzed and predicted the target of differentially expressed lncRNAs and CircRNAs(DELncRNAs; DECircRNAs).

## 2 Materials and methods

### 2.1 Experimental animals and reagents

The 30 SD female rats used in this experiment were eight weeks old and provided by Beijing Vitonglihua Experimental Animal Technology Co., LTD. SPF Grade rat maintenance feed (10kg/bag) purchased from Beijing Keao. The rats were fed adaptively after entering the barrier environment for one week. After staining the vaginal smears of 30 rats for ten days with the crystal violet staining method (source leaf: BYBZ14829), the rats were randomly divided into three groups: A, B, and C after confirming that each rat had a normal estrous cycle. Cyclophosphamide(CTX) was used to mold groups A and B. (source leaf: BYBZ30088). The modeling method was as follows: CTX50 mg/kg was injected once, and a maintenance dose of 8 mg/kg/d was injected continuously for 15 days. Group C received an equal volume of regular saline injection as a control. The pinhole was disinfected with a sterilized iodophor after each intraperitoneal injection. The estrous cycle of each group was observed again for ten days after completed the model. The rats whose estrous processes were disrupted and did not return to regular estrous cycles after ten days of vaginal smears were successful models.

### 2.2 Animal experimentation and specimen collection

Composition of the drug: Glehnia littoralis 18 g*、Ophiopogon Japonicus* 30 g*、*Rehmannia glutinosa 30 g*、Polygonatum Odoratum* 9 g. The four herbs listed above are all derived from the roots of specific plants. The Pharmacy Department of Shandong University of Chinese Medicine's Affiliated Hospital provided all drugs. MPNS (http://mpns.kew.org) verified all drug names. TCM compound was decocted by the Pharmacy Department of Shandong University of Chinese Medicine's Affiliated Hospital. The solvent was 500 ml of distilled water, and the decoction was 30 min. Each TCM dose was finally concentrated to 100 ml, with a drug concentration of 0.87 g/ml. The dose of YWD for rats should be 7.830 g/kg/d, according to the equivalent dose ratio table of human and animal body surface area. A and B groups were intragastrically irrigated around YWD, while C was irrigated intragastrically with an equal volume of normal saline. A simple random sample was used to choose three rats from each group. Isoflurane (Ryward: HYT11281368) was used to sedate the rats for 5 min. Following anesthesia, the rats were attached to the fixator, and their bilateral ovaries were removed. The rats were sacrificed by decapitation after the ovaries were removed.

### 2.3 RNA extraction

Total RNA was extracted from tissue using Plant RNA Purification Reagent (Invitrogen) according to the manufacturer's instructions, and genomic DNA was removed using rDNase I RNase-free (Takara). The ND-2000 and a 2100 Bioanalyzer (Agilent Technologies, Santa Clara, CA, USA) were used to assess RNA quality (NanoDrop Technologies). For the sequencing library, we utilized only high-quality RNA samples with a good optical density (OD260/280 = 1.8–2.2, OD260/230 ≥ 2.0, RIN≥8, 28S:18S ≥ 1.0, >10 μg).

### 2.4 Library construction and sequencing

The RNA-seq transcriptome strand library was prepared using 5 μg of total RNA using Illumina's TruSeqTM and honest RNA Kit (San Diego, CA). To summarize, Ribo-Zero Magnetic kit was used to deplete ribosomal RNA instead of poly(A) purification and then fragmented by fragmentation buffer first. Then, using random hexamer primers, first-stranded cDNA was synthesized. The RNA template was then removed, and a replacement strand was synthesized with dUTP instead of dTTP to generate ds cDNA. Because the polymerase did not incorporate past this nucleotide, incorporating dUTP quenched the second strand during amplification. The ds cDNA was separated from the second strand reaction mix using AMPure XP beads. In order to avoid the joining of these rough fragments during the adapter ligation process, a single ‘A' nucleotide was inserted at the 3′ ends. Finally, the ends of the ds cDNA were ligated with multiple indexing adapters. On 2% Low Range Ultra Agarose, libraries were size selected for cDNA target fragments of 200–300 bp, which were then PCR amplified for 15 PCR cycles with Phusion DNA polymerase. TBS380 quantified the paired-end RNA-seq sequencing library before being sequenced with the Illumina HiSeq xten/NovaSeq6000 (2150bp read length). Furthermore, TruseqTM Small RNA sample prep Kit was used to ligate 3 μg of total RNA with sequencing adapters (Illumina, San Diego, CA, USA). Then, cDNA was synthesized via reverse transcription and amplified via 12 PCR cycles to create libraries.

### 2.5 Read mapping and transcriptome assembly

The raw paired-end reads were trimmed and quality controlled using the default parameters of SeqPrep [14] (https://github.com/jstjohn/SeqPrep) and Sickle (https://github.com/najoshi/sickle). The clean reads were aligned to the reference genome in orientation mode using the HIASAT software [15](https://ccb.jhu.edu/software/hisat2/index.shtml). StringTie (https://ccb.jhu.edu/software/stringtie/index.shtml?t=example) was used to assemble the mapped reads of each sample in a reference-based approach [16].

### 2.6 LncRNA identification

Transcripts that overlapped with known protein-coding genes on the same strand, transcripts with a fragment count of 3, transcripts shorter than 200 nt, open reading frames longer than 300 nt, and exon numbers less than two were discarded. Using the Cufflinks suite's cuff-compare program, lncRNAs were classified as intergenic, intragenic, or antisense. Then we filtered transcripts with coding potential using the Coding Potential Calculator, Coding-Non-Coding Index, and Coding Potential Assessment Tool. Pfam Scan excluded the remaining transcripts with known protein domains based on Pfam HMM. The transcripts that remained were thought to be consistently expressed lncRNAs. We also use GREENC [17] (http://greenc.sciencedesigners.com/) to refer to LncRNAs. The FRKM method [18] was used to calculate the expression level of each LncRNA. |log2FC| >1 and p-value <0.05 by DEseq was used to extract differential expression LncRNAs(DELncRNAs) that were significant.

### 2.7 CircRNA identification

To identify CircRNAs, the CircRNA Identifier tools(CIRI) were used. It scans SAM files twice and collects enough information to identify and characterize CircRNAs. CIRI detects junction reads with PCC signals that reflect a CircRNA candidate during the first scanning of SAM alignment. For the links, preliminary filtering is implemented using paired-end mapping and GT-AG splicing signals. CIRI scans the SAM alignment again after clustering junction reads and recording each CircRNA candidate to detect additional hub reads and, in the meantime, performs further filtering to eliminate false positive candidates caused by incorrectly mapped reads of homologous genes or repetitive sequences. Finally, identified CircRNAs are output along with annotation data. The Spliced Reads per Billion Mapping method was used to calculate the expression level of each CircRNA. Differential expression CircRNAs(DECircRNAs) were discovered in the same way that DELncRNAs were.

### 2.8 Network pharmacology

BATMAN - TCM database (http://bionet.ncpsb.org.cn/batman-tcm/index.php) was used to obtain traditional Chinese medicine's active ingredients and action targets. Once all the targets were identified, any duplicates were eliminated.

### 2.9 Data classification and analysis

MSTEG + Gene numbering + transcriptome numbering was used to identify newly discovered LncRNAs. Newly discovered circles are named using chromosome numbering + sequence starting position + line ending posit. The volcano map was used to display the data after obtaining the differential expression data between the groups, and the DEncRNAs at the intersection of AvsB, BvsC, and AvsC were labeled in the map. Heat maps were used to show the expression of LncRNAs and CircRNAs with P-adjusted＜0.05 to improve the reliability of the differential expression data.

The Pearson correlation coefficient was used to calculate the co-expression of lncRNAs and mRNAs, CircRNAs and mRNAs, LncRNAs and miRNAs, CircRNA and miRNAs, and mRNAs and miRNAs when building the CeRNA regulatory network. If the correlation coefficient is more significant than one, p＜0.05 is positive; if it is less than one, p＜0.05 is negative. Attachment 1 details the mRNA and miRNA expressions of groups A and B used in the calculation. CircRNAs containing the intersection of AvsB, BvsC, and AvsC were used in Venny, and miRDB [19] (https://mirdb.org/) was used to predict miRNA binding. After that, the sequence combination of CircRNAs and miRNAs was scored with miRanda.v3.3a [20]. To calculate the energy required for possible base complementary pairing, enter score = 140 and energy = −1.

Using Venny2.1.0; the obtained YWD targets were intersected with LncRNA and CircRNA host genes. Host genes are significant genes that contain ncRNA transcription genes. To demonstrate the relationship between the active ingredient and host gene in regulating ncRNA, a network diagram of traditional Chinese medicine, active ingredient, and target gene was constructed using Cytoscape3.8.2.

## 3 Results

### 3.1 1Analysis of differential expression

**Fig. 1:**
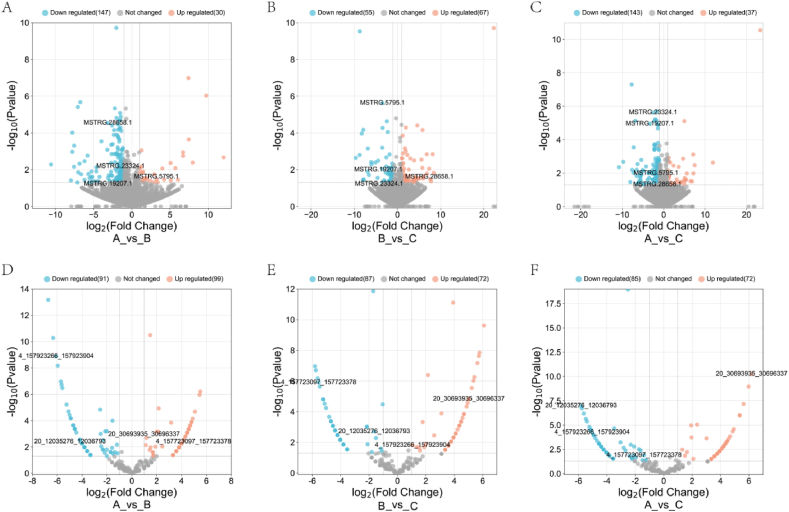
The expression patterns of DELncRNAs and DECircRNAs in comparisons of AvsB, BvsC, and AvsC. Light blue dots represent downgrades, while light orange dots represent upgrades. DELncRNA group comparisons are represented by A, B, and C, while DECircRNA group comparisons are represented by D, E, and F. Each graphic includes annotated comparisons between groups. A, B, and C in the diagram indicate the three groupings. The p-value means the significance of the difference between the two groups. Fold Change represents the fold difference in gene expression between the two groups. DELncRNAs and DECircRNAs are labeled in the figure because they appear in all three sets of differential expression comparisons.

**Fig. 2:**
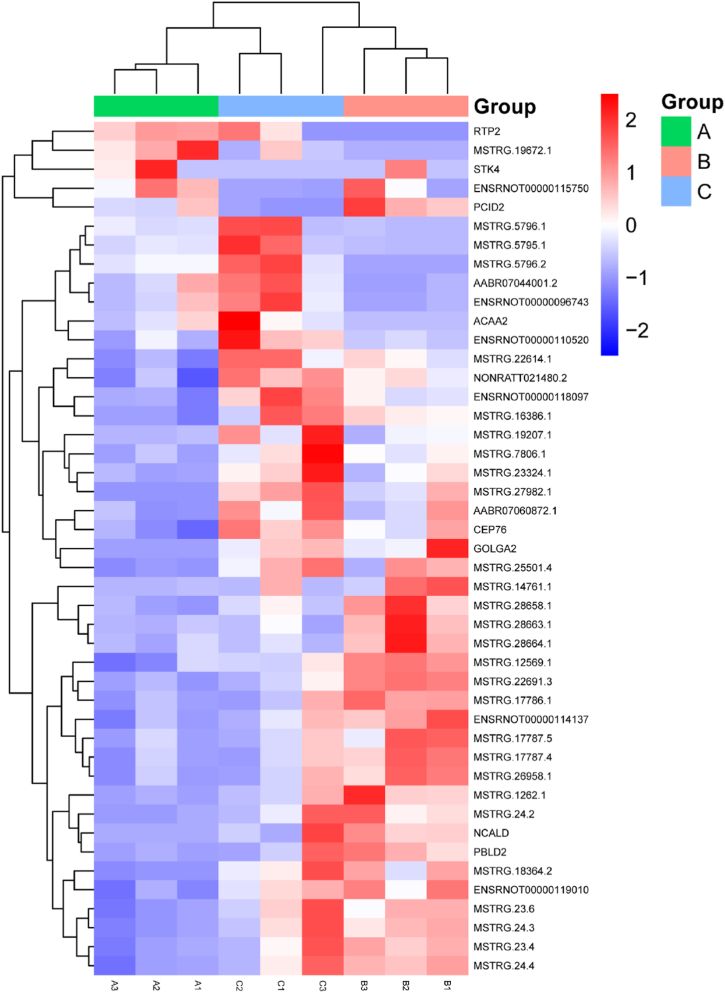
Rectangular heat maps were used to display 45 DELncRNAs with P-adjust＜0.05. Red represents high expression, while blue represents low expression. A, B, and C in the diagram indicate the three groupings. The lines on the left and top of the image show the cluster analysis results between the nine samples and Lncrnas.

**Fig. 3:**
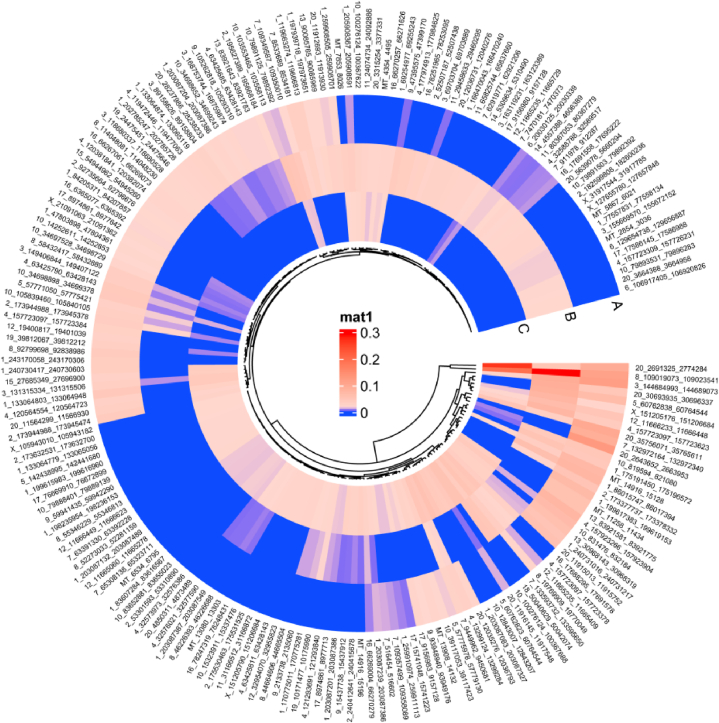
A ring heat map was used to display 190 DECircRNAs that met P-adjust＜0.05. Red represents high expression, while blue represents low expression. A, B, and C indicate the mean expression of samples from the three groups. The black wire lines in the circular image show the findings of the CircRNA cluster analysis.

In our study, 332 DELncRNAs were obtained statistically (P＜0.05), with AvsB： 177, BvsC：122, and AC：180. There were four intersection DELncRNAs in the three groups. We also get 311 DECircRNAs, with AvsB:190, BvsC:159, and AC:157. There were four intersection DECircRNAs in the three groups. Fig. 1 depicts the DEncRNAs of each group. Where D, E, and F are the expression of DECircRNAs and A, B, and C are DELncRNAs. Attachment 2 displays the differential expression data for LncRNAs and CircRNAs. After P-adjust＜0.05, 45 DELncRNAs and 190 DECircRNAs were obtained, and the expression details were shown in heat Fig. 2, Fig. 3. The findings show that YWD may affect these differentially expressed ncRNA.

### 3.2 CeRNA regulatory network construction

**Fig. 4:**
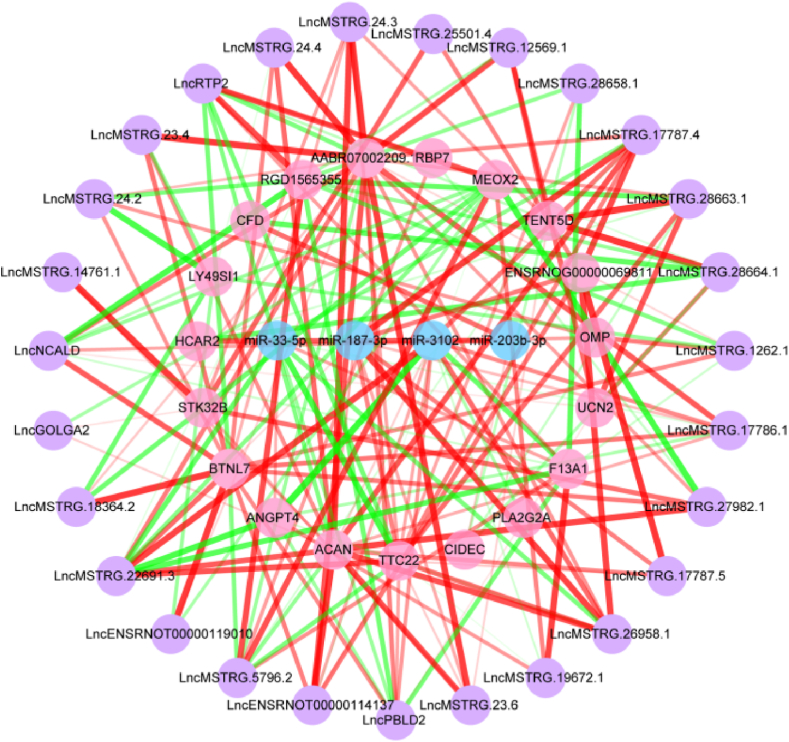
In the CeRNA network of LncRNA-miRNA-mRNA, blue represents miRNAs, red represents mRNAs, and purple represents LncRNAs. The thickness and color of the connection lines between nodes represent the connection's P value. The green connection line indicates a negative correlation, while the red line indicates a positive correlation.

**Fig. 5:**
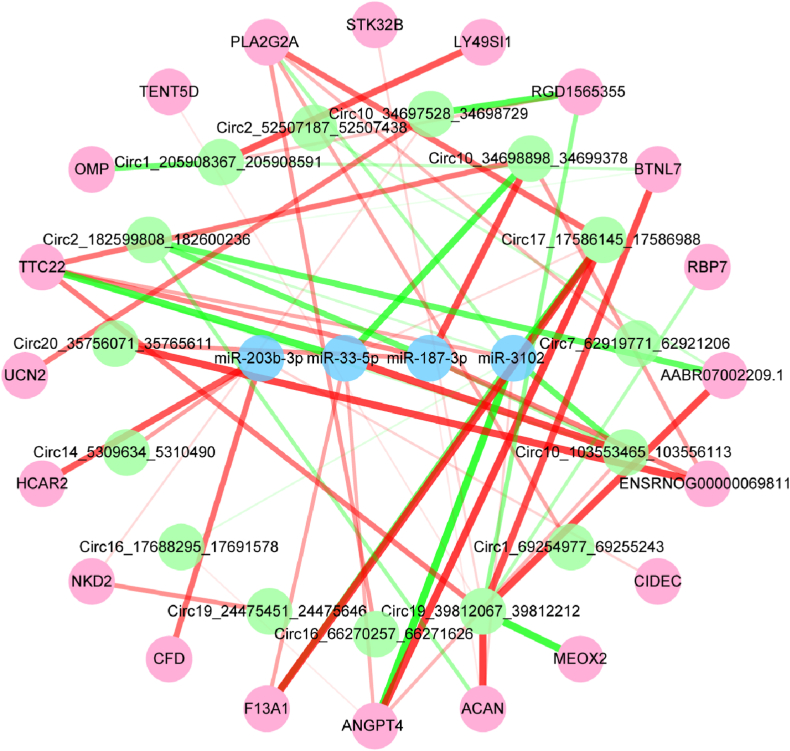
Blue represents miRNAs, red represents mRNAs, and green represents CircRNAs in the CeRNA network CircRNA-miRNA-mRNA. The wiring is identical to that shown in Fig. 4.

Following the calculation of the co-expression relationship, P＜0.01 was used as the screening network control condition, and the network was displayed using Cytoscape 3.8.2. Fig. 4 depicts the CeRNA network of LncRNA-miRNA-mRNA, which includes 27 LncRNAs, 4 miRNAs, and 19 mRNAs. Fig. 5 shows the CeRNA network of CircRNA-miRNA-mRNA, which consists of 15 CircRNAs, 4 miRNAs, and 20 mRNAs. Together, these results suggest that LncMSTRG.22691.3/miR-3102/ANGPT4 and 1Circ10 34698898 34699378/miR-33-5p/TTC22 were the highly correlated regulatory networks.

### 3.3 CircRNAs as a cavernose-binding miRNAs prediction

**Fig. 6:**
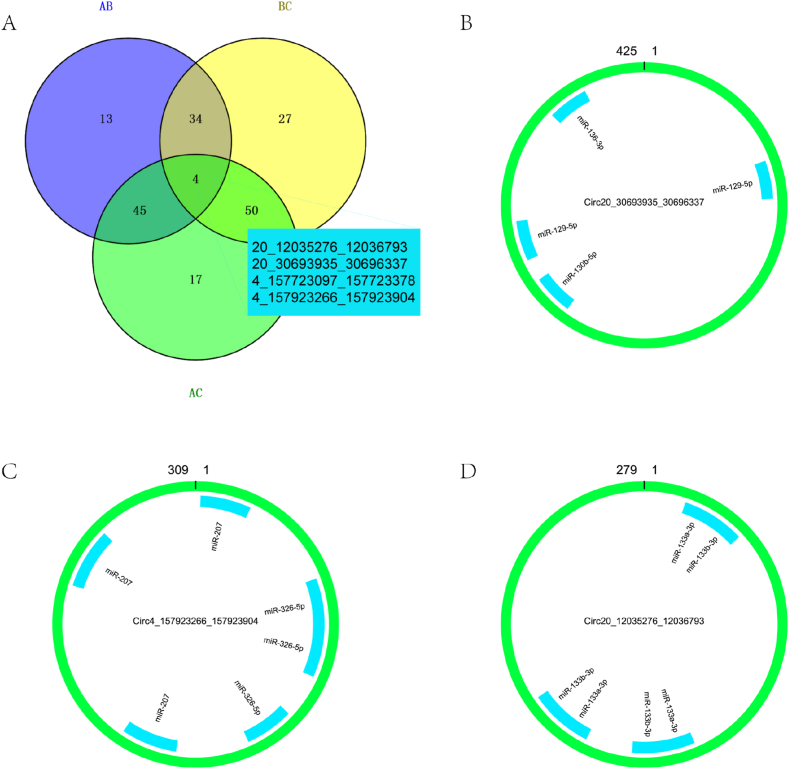
A represents DECircRNAs at the intersection of AvsB, BvsC, and AvsC. B, C, and D represent the binding sites of CircRNAs and miRNAs, respectively. The green rings in B, C, and D represent CircRNAs, and the inner blue rectangles are miRNAs. The length of the CircRNA base sequence is shown by the number in the green ring superscript.

**Fig. 7:**
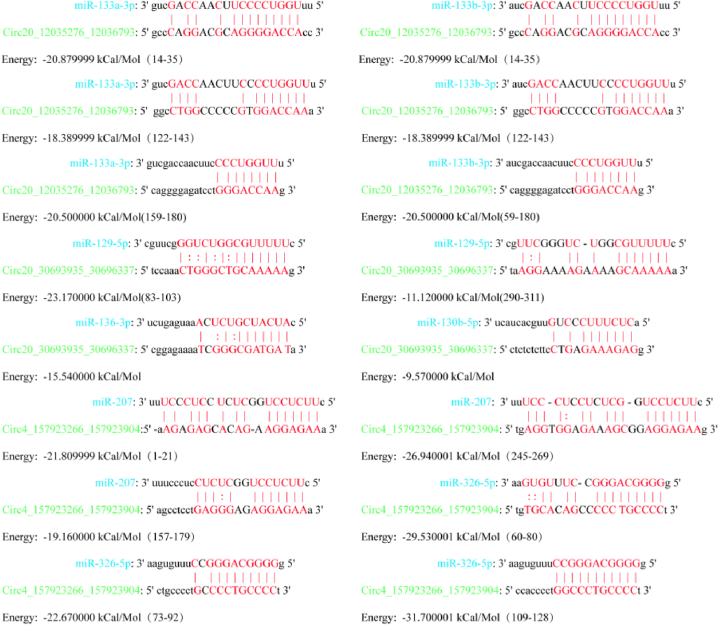
The precise location of CircRNAs binding to miRNAs and the details of base complementarity are depicted. "|" represents the hydrogen bond, ":" means the two hydrogen bonds and energy represents the energy required for base pairing. Red represents sequences with complementary bases.

Venny2.1.0 identified four DECircRNAs at the intersection of AvsB, BvsC, and AvsC. Fig. 6A provides more information. The combination of miRDB prediction target and miRanda.v3.3a sequence showed that Circ20_12035276_12036793 can bind to 2 miRNAs, Circ20_30693935_30696337 can bind to 3 miRNAs, and Circ4_157923266_157923904 can bind to 2 miRNAs. There were no targeted miRNAs predicted for Circ4 157723097 157723378. Fig. 6, Fig. 7 show the sequence combination in detail. Attachment 3 contains detailed information about each sequence binding site.

### 3.4 Active ingredient-target relationship

**Fig. 8:**
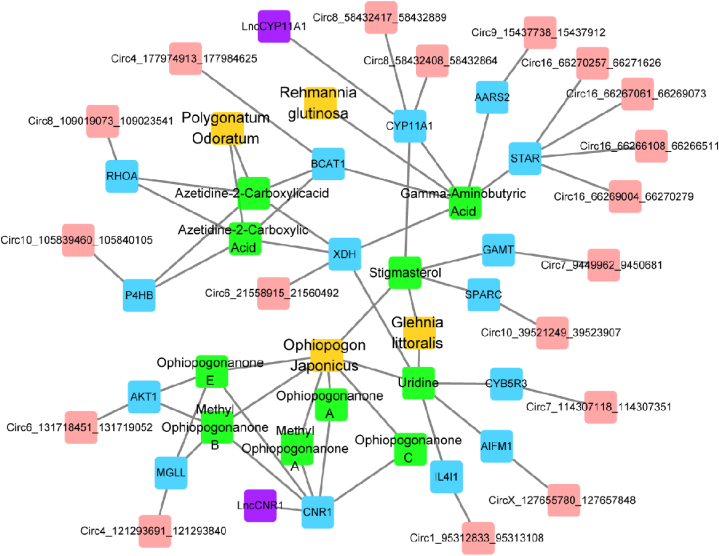
TCM-active ingredients-target-ncRNAs: TCM is represented by orange; The color green represents the active ingredient; The intersection of the YWD target and the host gene of ncRNA is represented in blue; The light red represents CircRNAs; LncRNAs are represented by purple.

Through analysis and summarization, a total of 519 YWD targets were obtained. 15 key targets and 10 active components were discovered via intersections with host genes of DELncRNAs and DECircRNAs. See Fig. 8 for details.

## 4 Discussion

In this study, we examined the transcriptomic differences in ncRNA expression in POI rats affected by YWD, as well as the relationship between the expression of DEncRNAs and the expression of differentially expressed genes, revealing the mechanism of action of YWD intervention in premature aging. We also discovered numerous new LncRNAs and CircRNAs. The existing CircRNA database, in particular, only contains data from humans and mice.

The role of LncNCALD, LncGOLGA2, LncPBLD2, and newly discovered LncRNAs and CircRNAs after YWD intervention in POI rats is unknown. We only know what these ncRNA-related genes do right now. For example, low NCALD expression protects against myeloid muscle atrophy, while overexpression promotes apoptosis [21,22]. GOGLA2, a gene involved in cell proliferation and autophagy [23], has been linked to some neuromuscular diseases and is essential for human development [24]. Some genes studied in the regulatory network are directly related to follicle development——For example, LncMSTRG.19672.1-miR-3102-F13A1, LncMSTRG.22691.3-miR-33-5p-ANGPT4, and Circ10_34698898_34699378-miR-187-3p-UCN2. When the expression of protein in the follicular fluid of mature and immature bovine follicles was compared, F13A1 was found to be highly expressed in the follicular fluid of mature bovine follicles, indicating that F13A1 may be involved in follicular maturation [25]. ANGPT4 is involved in granulosa cell regulation during follicular development [26]. UCN2 overexpression in the ovary reduces steroid production and inhibits follicle maturation [27].

CircRNAs can act as sponge molecules for miRNAs, reducing miRNA degradation and weakening miRNA regulation [28]. Three of the four intersection DECircRNAs predicted by us predicted possible binding miRNAs in miRDB. The three DECircRNAs had good sequence binding with miR-133a-3p, miR-129-5p, miR-136-3p, miR-326-5p, miR-133b-3p, miR-130b-5p, and miR-207. Most of these microRNAs are involved in cell proliferation, apoptosis, and follicle development. MiR-133a-3p, for example, can promote granulosa cell proliferation while inhibiting apoptosis in geese by targeting ANSO1 [29]. MiR-129-5p was discovered to be involved in follicular development by comparing the differentially expressed miRNAs during follicular growth in sheep [30]. MiR-136-3p can influence the growth of ovarian granulosa cells by regulating the downregulation of the luteinizing hormone receptor [31]. MiR-130b-5p can inhibit the secretion of pro-inflammatory cytokines and reduce the damage caused by microglia cells to neuronal cells [32]. MiR-207 inhibits autophagy and promotes cardiomyocyte apoptosis by inhibiting the expression of LAMP2, which is involved in developing type 2 diabetic cardiomyopathy [33]. Furthermore, we use network pharmacology technology to identify and obtain active ingredients that may regulate LncRNA and CircRNA, such as Stigmasterol, Uridine, Ophiopogonanone A, Gamma-Aminobutyric Acid, and others. The majority of these components have antioxidant properties [34,35]. Although the role of these ncRNAs is unknown, the relationship we discovered aids in elucidating the molecular mechanism of action of YWD treatment of POI.

## 5 Conclusion

Our research enriched the molecular mechanism of YWD treatment of POI at the transcriptomic level, discovering 45 DELncRNAs and 190 DECircRNAs of high research significance. By comparing the DEncRNAs in groups A and B, two CeRNA regulatory networks were built. Targeted miRNAs were also predicted for three groups of intersecting DECircRNAs. These findings can serve as a foundation for future research to confirm the molecular role of YWD in the treatment of POI.

## Ethics approval

All animal use has been approved by the Animal Welfare Ethics Review Committee of Shandong University of Traditional Chinese Medicine with the approval number SDUTCM20210224024.

## Funding

This work was supported by the 10.13039/501100007129Natural Science Foundation of Shandong Province (ZR2021MH404; ZR2022MH163).

## Author contribution statement

Weisen fan: Conceived and designed the experiments; Wrote the paper.

Yingjie zhang: chen wang: Contributed reagents, materials, analysis tools or data.

Dandan wang: Performed the experiments.

Jie yang: Analyzed and interpreted the data.

## Data availability statement

Data will be made available on request.

## Additional information

No additional information is available for this paper.

## Declaration of competing interest

The authors declare that they have no known competing financial interests or personal relationships that could have appeared to influence the work reported in this paper.

## Abbreviations

YWD: yiwei Decoction
TCM: traditional Chinese medicine
LncRNA: long noncoding RNA
CircRNA: circular RNA
POI: premature ovarian insufficiency
NcRNA: non-coding RNA
CTX: cyclophosphamide
DELncRNAs: differential expression LncRNAs
DECircRNAs: differential expression CircRNAs
CIRI: CircRNA Identifier tools
NCALD: neurocalcin-delta
GOLGA2: Golgin subfamily A member 2
PBLD2: Phenazine biosynthesis-like domain-containing protein 2
F13A1: Coagulation factor XIII A chain
ANGPT4: Angiopoietin-4
UCN2: Urocortin-2
ANOS1: Anosmin-1
LAMP2: Lysosome-associated membrane glycoprotein 2
